# Organisational and Team-Level Strategies to Enhance Work Engagement and Mitigate Burnout Among Nurse Case Managers: A Global Scoping Review with Implications for the Gulf Region

**DOI:** 10.3390/nursrep16040145

**Published:** 2026-04-17

**Authors:** Ahmed Yahya Ayoub, Carin Maree, Neltjie van Wyk

**Affiliations:** 1 Department of Nursing, Case Management, Sheikh Shakhbout Medical City, Pure Health, Abu Dhabi 11001, United Arab Emirates; 2 Department of Nursing Science, Faculty of Health Science, University of Pretoria, Pretoria 0084, South Africaneltjie.vanwyk@up.ac.za (N.v.W.)

**Keywords:** burnout, professional, case management, case managers, nurses, patient care team, work engagement

## Abstract

**Introduction:** Work engagement among nurse case managers is central to safe, efficient, person-centred care, yet organisational and team-level factors that support engagement or mitigate burnout remain poorly synthesised. **Aim:** To map organisational and team-level strategies that enhance work engagement or reduce burnout among nurse case managers and aligned roles, as well as to consider their applicability to Gulf health systems. **Method:** We conducted a scoping review in accordance with the Arksey and O’Malley framework as refined by Levac et al. and reported it in line with PRISMA-ScR and PRISMA-S guidance. Six databases and targeted sources were searched for English-language records published between 2015 and 2025. Two reviewers independently screened titles/abstracts and full texts against predefined eligibility criteria, charted data using a piloted form, and synthesised findings thematically against Job Demands–Resources (JD-R) domains. **Results:** Of 303 records identified, 248 were screened after deduplication, and 11 studies were included. Across nine health systems, findings were mapped to three JD-R domains: job resources, job demands, and personal resources. The most recurrent resource-related strategies involved structural supports, staffing stability, coordination infrastructure, and supportive leadership or team practices. Key demands included role complexity, high caseloads, coordination workload, discharge pressures, and staffing instability. Personal-resource approaches were fewer and mainly involved stress management, communication, and reflective practice interventions. Engagement was infrequently measured directly, and only one empirical intervention study originated from a Gulf health system. **Conclusions:** This JD-R-informed scoping review suggests that strengthening structural, staffing, and coordination resources, alongside supportive leadership and team climates, may be important for sustaining engagement and limiting burnout among nurse case managers. However, these findings should be interpreted as exploratory signals that map the current evidence landscape rather than definitive evidence of effectiveness. Multi-component JD-R-informed bundles in Gulf region health systems should therefore be prioritised for context-sensitive co-design, piloting, and evaluation.

## 1. Introduction

Work engagement among nurses is increasingly recognised as a strategic lever for safe, high-quality care, efficient services, and workforce sustainability. Engagement is commonly defined as a positive, fulfilling work-related state characterised by vigour, dedication, and absorption, which is conceptually distinct from burnout and predicts important individual and organisational outcomes in healthcare settings [1,2,3]. Evidence syntheses link higher staff engagement to stronger patient safety culture and fewer reported adverse events, while nurse burnout is consistently associated with lower care quality, reduced safety, and poorer patient satisfaction [4]. Recent multi-country and single-system studies additionally show that engagement relates to patient satisfaction and safety and to nurse retention and performance outcomes [5]. Because engagement and burnout are shaped by the balance between work demands and available resources, these constructs are especially important in roles characterised by sustained coordination, relational work, and cross-setting accountability. Burnout is commonly understood as a work-related syndrome characterised by emotional exhaustion, depersonalisation or cynicism, and reduced professional efficacy or personal accomplishment [6].

Nurse case managers occupy a pivotal position at the interface of clinical, administrative, and social care functions. Their role typically includes assessment, planning, coordination, interprofessional communication, advocacy, and ongoing monitoring across episodes and settings of care [6]. The breadth of these responsibilities exposes case managers to substantial job demands, including heavy caseloads, complex navigation of services, emotional labour, documentation requirements, and legal or regulatory tasks that can accumulate into chronic stressors if poorly managed [6]. In integrated and person-centered models, nurse case managers also lead collaborative processes that require sustained cognitive, relational, and coordination effort, underscoring the need for organisational and team resources that enable effective and sustainable practice [7].

**Figure 1:**
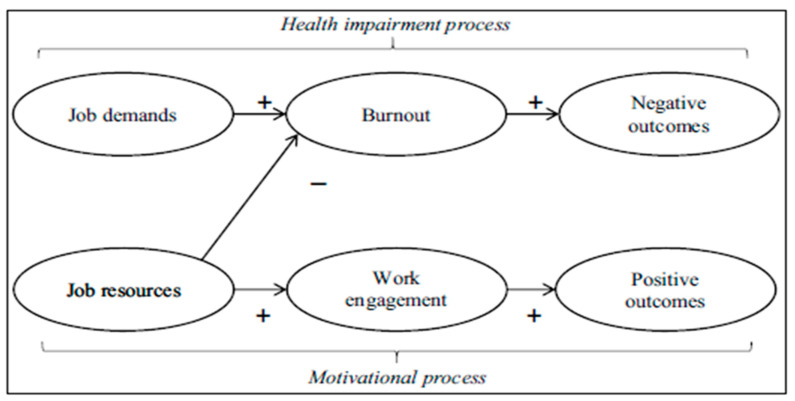
Job Demands–Resources (JD-R) Model [8].

The Job Demands–Resources (JD-R) theory provides a robust framework for explaining how characteristics of work shape burnout and engagement. Figure 1 summarises the model. In this model, job demands are the physical, psychological, social or organisational aspects of work that require sustained effort and are therefore associated with physiological or psychological costs, whereas job resources are aspects of the job that help workers achieve work goals, reduce the impact of demands or stimulate learning and development, such as autonomy, support, feedback and adequate staffing. Personal resources, including self-efficacy and coping skills, are also recognised as relevant to how workers experience their jobs. The health impairment process links excessive or poorly managed demands to exhaustion and negative outcomes, whereas the motivational process links adequate resources to engagement and positive outcomes [8]. Contemporary formulations emphasise that resources can buffer the impact of demands and that resources are the most proximal drivers of engagement [9,10]. Meta-analytic evidence using the JD-R framework supports these propositions and identifies consistent antecedents and outcomes of engagement across occupations, including healthcare [11]. In nursing specifically, studies confirm the operation of the JD-R processes, with job demands predicting burnout and job resources predicting engagement and better work outcomes [12].

For nurses, structural demands such as staffing shortfalls, high patient-to-nurse ratios, long or rotating shifts, and escalating documentation contribute to burnout risks and degraded outcomes. Systematic reviews and large observational studies demonstrate that higher registered-nurse staffing is associated with lower mortality and better patient outcomes, while understaffing and 12 h shifts correlate with higher burnout and dissatisfaction [13]. Reviews and meta-analyses further show associations between nurse burnout and poorer quality and safety, and they document widespread moderate-to-high burnout levels across settings [14]. Schedule characteristics and workload intensity are repeatedly implicated, and schedule flexibility appears protective [15].

Conversely, organisational and team resources are reliably linked to engagement and well-being. Positive leadership styles, including transformational, engaging, and caring leadership, show consistent associations with higher nurse engagement and better well-being [16]. Perceived organisational and supervisory support are inversely related to turnover intention and directly related to engagement [17]. Team climate also matters: effective teamwork and psychological safety enable speaking up, learning, and coordination, and are associated with improved patient and organisational outcomes as well as more positive, engaging work environments [18]. Work environment programmes, such as Magnet recognition, which institutionalise participatory decision-making, supportive supervision, and professional autonomy, are linked in many studies to lower burnout, higher satisfaction, and in several syntheses to better patient outcomes [19].

Intervention evidence on organisational and team-level strategies to enhance work engagement and reduce burnout among nurse case managers and related nursing roles is growing. Organisation-directed approaches that modify structures, leadership practices, workflows, or staffing show benefits for workforce outcomes, although effect sizes vary and durability is mixed [20]. Randomised and quasi-experimental studies report that job-crafting programmes can elevate engagement, and leadership development initiatives can strengthen unit-level management capabilities that underpin team climate and engagement [21]. Reviews on burnout interventions among nurses identify mindfulness and multimodal programmes among the most frequently tested strategies, with small to moderate improvements in burnout and related outcomes [22]. Broader healthcare worker reviews similarly find that workplace interventions can improve engagement and resilience and reduce burnout, while highlighting methodological limitations and the need for context-sensitive organisational designs [23].

The Gulf region context heightens the relevance of organisation- and team-level strategies. Synthesis of studies from Gulf Cooperation Council countries during and after the pandemic documents substantial mental health burdens among healthcare workers, including stress, anxiety, depression, and burnout [24]. Recent multicentre work from Saudi Arabia and the United Arab Emirates shows notable burnout prevalence among nurses in private tertiary hospitals and identifies workload, staffing, and administrative support as salient factors [25]. Additional studies from the region report high psychological distress, moral distress, and intention to leave among nurses, underscoring the need for local organisational responses that strengthen engagement and retention [26].

Despite the expanding literature on nursing engagement and burnout, two gaps remain. First, most syntheses treat nursing as a single occupational group, with limited attention to nurse case managers whose roles combine clinical coordination, navigation, and administrative accountability, as well as those who may, therefore, face a distinct profile of demands and resources [27]. Second, evidence-based intervention often centers on individual-focused approaches rather than organisational and team-level strategies that align with the JD-R model’s emphasis on structural resources and the buffering of demands [23]. A focused scoping review that maps organisational and team-level strategies to enhance work engagement among nurse case managers, and that assesses the applicability of global evidence to Gulf region health systems, can therefore inform leadership, workforce, and service-delivery policy in similar contexts [28].

On this basis, the review was guided by the following research question: What organisational and team-level strategies have been reported to enhance work engagement or mitigate burnout among nurse case managers and closely aligned roles, and what do these findings suggest for adaptation in Gulf region health systems? Accordingly, this scoping review aimed to map and synthesise such strategies using the JD-R framework as an interpretive lens.

## 2. Methods

### 2.1. Design and Reporting

This scoping review was conducted and reported in accordance with the PRISMA-ScR (Preferred Reporting Items for Systematic Reviews and Meta-Analyses Extension for Scoping Reviews) guidelines. The search strategy was additionally reported in line with PRISMA-S recommendations. Prospective registration was not undertaken because the review was conceived as a scoping exercise to map a heterogeneous evidence base rather than to estimate pooled effects. Nevertheless, the eligibility criteria, search approach, and charting domains were specified a priori and are reported in full to support methodological transparency.

**Figure 2:**
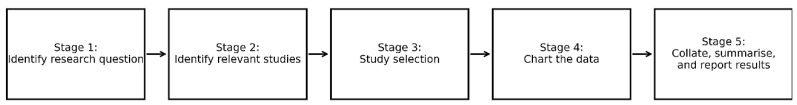
Five-stage scoping review framework [29].

We conducted a scoping review guided by the five-stage framework articulated by Arksey and O’Malley [29] and refined by Levac and colleagues [30], with reporting aligned to PRISMA-ScR and search reporting to PRISMA-S (framework overview in Figure 2).

### 2.2. Eligibility Criteria

Eligibility followed a PCC schema including study designs, timeframe, language, outcomes and availability of the full text of the literature, as illustrated in Table 1 below. The 2015–2025 window was chosen to capture contemporary evidence relevant to current case-management roles, team-based care structures, and workforce conditions, while maintaining a feasible scope for a scoping review. Restriction to English-language publications was applied for feasibility and consistency of screening and data extraction; however, this may have excluded relevant studies from non-English-speaking settings and is acknowledged as a limitation.

### 2.3. Information Sources and Search Strategy

We searched MEDLINE via PubMed, CINAHL, Embase, APA PsycINFO, Scopus, and Web of Science Core Collection within the stated window. Strategies combined controlled vocabulary and keywords for role terms (“nurse case manager,” “nurse navigator,” and “care coordinator”), focal outcomes (“work engagement,” and “burnout”), JD-R constructs (“job demand*,” “job resource*,” and “JD-R”), and organisational or team terms (“program*,” “policy,” “organisation*,” and “team*”). Strategies were translated across databases and underwent peer review by an information specialist using the PRESS 2015 checklist prior to execution. Full search strategies and the limits employed were in line with the PRISMA-S framework as described by Rethlefsen et al. [31]. The searches were executed on 15th of January 2026, and all results were limited to records published between 1 January 2015 and 31 December 2025. The full database-specific search strategies, including controlled vocabulary, free-text terms, Boolean combinations, and limits, are provided in Supplementary Table S1.

### 2.4. Record Management and Study Selection

**Figure 3:**
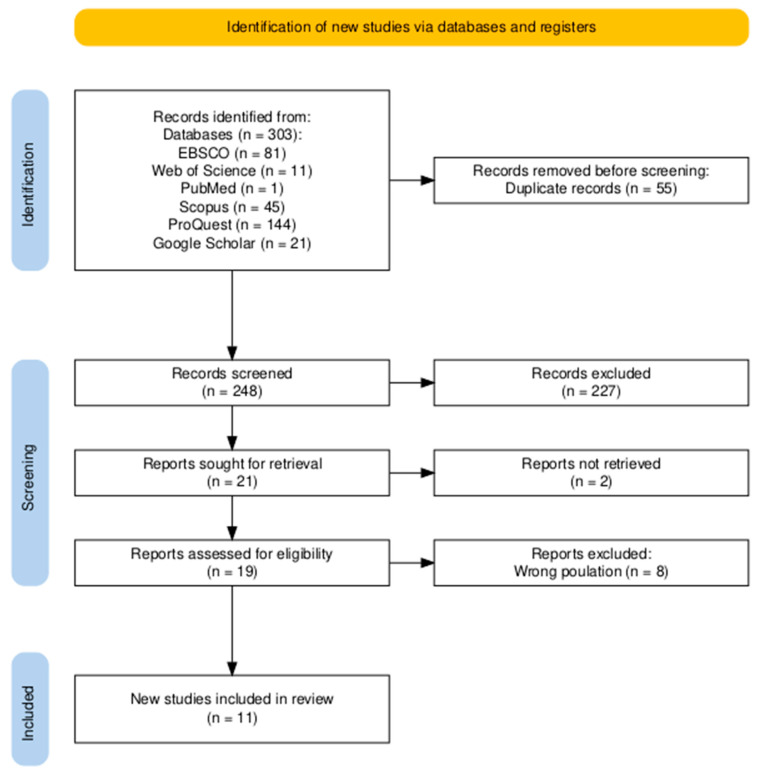
PRISMA-ScR flow of study selection.

All records were exported to Rayyan (v2025) and initially de-duplicated. Titles and abstracts were screened independently by two reviewers in Rayyan, followed by full-text assessment against the criteria after a pilot calibration. Disagreements were resolved by discussion, and on a few occasions, a third reviewer’s adjudication was sought. Reasons for full-text exclusion were logged, and the selection process was summarised in a PRISMA-ScR flow diagram (Figure 3) adapted from [32].

### 2.5. Data Charting

**Figure 4:**
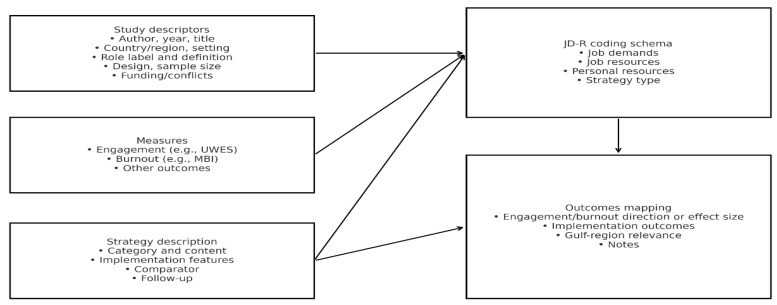
Data-charting domains and JD-R coding schema.

We developed and piloted a structured charting form before full extraction. One reviewer charted all items, and a second reviewer independently verified a random 30 percent sample. We extracted the following: bibliographic details; country and setting; role description; study design and sample; measures of engagement and burnout; reported job demands and job resources; organisational or team strategy content and implementation features; comparators where applicable; outcomes and effects; theoretical framing; and funding. A representation of the charting variables is shown in Figure 4, which summarises the data-charting domains and JD-R coding schema.

### 2.6. Operational Definitions and JD-R Coding

The Job Demands–Resources (JD-R) framework was applied as an interpretive framework for charting, organising, and synthesising the review findings; it was not assumed to structure the design, measurement, or reporting of the included primary studies. Within this framework, job demands were defined as aspects of work requiring sustained effort and incurring psychological or physiological cost, whereas job resources were defined as aspects that help achieve work goals, reduce demands, and foster growth and development. Each strategy was classified as primarily demand-reducing, resource-enhancing, or personal-resource cultivating, and we noted where resources appeared to buffer demand effects [10].

### 2.7. Synthesis and Mapping

We summarised study and strategy characteristics with descriptive statistics and mapped strategies by organisational level (system, facility, unit) and function (leadership practices; staffing and workload design; team climate and psychological safety; training and mentoring; workflow and digital tools). We then synthesised mechanisms, implementation considerations, and reported effects, and highlighted applicability to Gulf region health systems. This section also cross-references the PRISMA flowchart (Figure 3) for transparency about included evidence. Because the aim was to map the evidence landscape, eligible records included empirical studies, as well as developmental, protocol-based, and review-level contributions. These were synthesised together for mapping purposes, but they were interpreted differently: empirical studies informed outcome-related observations, whereas developmental, protocol-based, and review-level records informed conceptual, design, and implementation-related insights.

### 2.8. Critical Appraisal

We did not conduct a formal risk-of-bias appraisal because the objective was to map the breadth and nature of evidence rather than estimate pooled effects. Where authors reported quality assessments, these were noted to aid interpretation. Although a formal critical appraisal was not undertaken, variation in study design, measurement strategy, follow-up duration, and completeness of reporting was considered during interpretation of the evidence base.

### 2.9. Ethical Considerations

Ethical approval was not required because this scoping review synthesised previously published and publicly available reports and involved no interaction with human participants or access to identifiable data. We followed recognised publication-ethics practices, including transparent disclosure of funding and any conflicts of interest.

## 3. Results

### 3.1. Study Selection

Database searches produced 303 records. After removing 55 duplicates, 248 titles and abstracts were screened. We sought 21 full texts, could not retrieve 2, and assessed 19 for eligibility. Eight were excluded because they did not meet the population criteria, leaving 11 studies for inclusion (Figure 3; counts detailed in the PRISMA-ScR flow).

### 3.2. Characteristics of Included Evidence

The 11 records spanned nine health systems and roles aligned with nurse case management, including nurse navigators, care coordinators, registered nurse care managers, transitional care nurses and dementia care coordinators (Table 2). Designs included quasi-experimental and observational quantitative studies, mixed methods and realist evaluations, qualitative studies, one scoping review, one development report and one systematic review protocol. Burnout was most commonly measured with the Maslach Burnout Inventory, and work engagement with the Utrecht Work Engagement Scale. Accordingly, the included evidence comprised both empirical outcome-generating studies and non-outcome-generating contributions, including a development report and a systematic review protocol. In the narrative synthesis, empirical studies were used primarily to inform outcome-related interpretations, whereas the latter contributions were used to contextualise intervention design, implementation considerations, and evidence gaps. The included studies varied substantially in design, methodological robustness, and outcome reporting; findings should therefore be interpreted with appropriate caution. In many included studies, work engagement was not measured directly, and interpretation therefore relied partly on related constructs such as burnout, well-being, resilience, or job satisfaction. Within the JD-R framework, these constructs are conceptually related to engagement but are not interchangeable, and they were interpreted accordingly.

### 3.3. Thematic Synthesis Mapped to the JD-R Model

Findings are organised by strategies that reduce job demands, enhance job resources or cultivate personal resources, consistent with JD-R health impairment and motivational processes (Table 3). Table 3 presents the three JD-R domains as themes (job resources, job demands and personal resources) and the associated sub-themes, along with operational examples and the studies contributing to each sub-theme.

#### 3.3.1. Theme 1. Job Resources

Findings under the first JD-R domain described organisational and team-level resources that support nurse case managers and closely aligned roles. Six sub-themes were identified, corresponding to the sub-headings in Table 3

**Structural enablers**

Several studies highlighted underlying structural arrangements that create a more sustainable context for case management and coordination roles. In a realist evaluation of dementia care coordinators, workforce design, dedicated funding and clear governance arrangements were described as important conditions for sustaining the role in pressured systems [43]. A scoping review of nurse navigators recommended integrated team structures and permanent funding to address overload and inefficient workflows [36]. Hospital coordinators in South Korea emphasised institutional support from the host organisation as a facilitator for transitional care programmes, reinforcing that formal programme backing functions as a core structural resource [42].

**Coordination infrastructure**

Coordination infrastructure refers to tools and processes that reduce the transaction costs of care coordination. Hospital coordinators called for a shared information platform and institutional assistance to support cross-setting communication [42]. Dementia care coordinators reported benefits from having a single point of contact and linked information systems that allowed them to track patients across primary care networks [43]. In the cancer navigation context, navigators operated within systems that combined coordination, advocacy, education, psychosocial support and workflow improvement, underscoring the value of integrated care pathways and information flows as enabling infrastructure [36].

**Staffing stability and adequate full-time equivalent staffing**

Staffing arrangements emerged as another central job resource. In a large retrospective analysis of primary care teams, continuous churn in registered nurse care manager positions was associated with poorer access metrics, including longer waits for appointments, whereas team stability and adequate full-time equivalent levels were associated with better access outcomes [41]. This study indicates that staffing stability and sufficient staffing levels function as organisational resources that support both patient access and the sustainability of care manager roles.

**Training and supportive supervision**

Training and supervisory support were described as resources that can strengthen coordinators’ capacity to manage complex workloads. In integrated physical–behavioural care, care coordinators reported high perceived organisational support, including formal training and supportive supervisors, in settings where burnout risk was low [39]. A systematic review protocol on interventions to improve nurses’ work engagement planned to synthesise trials of resource building, leadership and health promotion interventions, reflecting an emerging focus on training and supervisory practices as levers for engagement [37]. Although this review has not yet reported results, it indicates that structured training and supportive supervision are recognised as potential JD-R resources in the nurse workforce.

**Manager-staff development**

Manager–staff development interventions were explicitly aligned with the JD-R framework in one programme. Guo et al. [35] described the development of a dualistic nurse-manager intervention built around Appreciative Inquiry, with six sessions across nine weeks designed to enhance resources and engagement. The programme was considered ready for feasibility testing, but had no outcome data at the time of reporting. In Brown et al. [40], nurse and midwife navigators participated in action learning groups alongside leadership and governance supports. Quantitative changes were limited, but qualitative accounts suggested that leadership attention and developmental structures contributed to sustained coping and resilience over time.

**Team-based resource building**

Team-based resource building captured collective practices that support case managers within multidisciplinary teams. A participatory-action intervention on older people’s wards sought to increase work engagement by enhancing social support and decision influence at the team level. Engagement scores did not improve, but mediation analysis supported the relevance of JD-R and self-determination mechanisms, and implementation readiness was reported as critical for change [34]. In integrated physical–behavioural care, coordinators reported strong team support and low burnout risk, suggesting that supportive team climates can operate as protective resources [39]. Brown et al. [40] described action learning groups that enabled navigators to reflect on challenges and share coping strategies, with qualitative data indicating benefits for well-being despite minimal quantitative change. Taken together, these studies describe structured team forums and peer support operating as job resources, even when measured effects on engagement or burnout are modest.

#### 3.3.2. Theme 2. Job Demands

The second JD-R domain comprised stressors that place a sustained load on nurse case managers and analogous roles. Five sub-themes were identified in line with Table 3.

**Role complexity**

Role complexity was reported as a core demand for navigators and care coordinators. In the multi-method study of nurse and midwife navigators, participants described broad and shifting responsibilities that spanned coordination, education, advocacy and administrative tasks, often accompanied by high documentation requirements [40]. These overlapping functions required substantial cognitive and relational effort and were perceived as demanding even when organisational supports were present.

**High caseloads and system tensions**

Increasing caseloads and system pressures were described as prominent demands in the realist evaluation of dementia care coordinators. Coordinators reported that rising case numbers, coupled with system-level constraints, created tensions that could threaten role sustainability and contribute to strain [43]. These pressures were experienced alongside the responsibility of acting as a bridge between services, which intensified the perceived demand when system capacity was constrained.

**Accelerated discharges and disrupted connections**

Accelerated discharges and disrupted continuity of care were evident in transitional care contexts. In a qualitative study of nurses’ experiences during the COVID-19 period in Japan, transitional care nurses described accelerated hospital discharges, disrupted connections with community providers and hybrid workflows as key challenges [38]. These changes compressed coordination time and complicated efforts to ensure safe continuity of care. Similar concerns about discharge pressures were reported by hospital coordinators involved in congenital long-term disease programmes [42].

**Cross-sector coordination workload**

Cross-sector coordination workload represented another recurrent demand. Care coordinators working in integrated physical–behavioural health services described complex navigation across sectors and settings as a central part of their role [39]. Hospital coordinators in South Korea reported heavy cross-institutional coordination workloads as a barrier to effective transitional care [42]. Transitional care nurses in Japan also highlighted cross-sector coordination tasks as taxing, especially when combined with pandemic-related disruptions [38].

**Team churn or vacancy**

Team churn and vacancy were described as demands that indirectly affect case manager roles. The retrospective analysis of registered nurse care managers showed that teams with ongoing churn or vacancy had poorer access outcomes, indicating that instability in key roles created additional workload and stress for remaining staff [41]. Repeated turnover and reliance on temporary staff can disrupt relationships with patients and colleagues, reduce continuity and require extra time for onboarding and coordination, all of which function as job demands.

#### 3.3.3. Theme 3. Personal Resources

The third JD-R domain captured strategies that aimed to build personal resources such as coping skills and reflective capacity. Two sub-themes correspond to the personal-resource entries in Table 3.

**Stress management and communication skills**

A quasi-experimental study in Saudi Arabia evaluated a two-day person-directed programme for hospital mental health nurses that combined stress management content with communication and social skills training [33]. Burnout scores improved at one month but partially rebounded by six months. Although conducted in a broader nursing population, the focus on managing emotional fatigue and communication challenges is relevant to nurse case managers who also work at the interface of complex clinical and relational demands.

**Self-care and reflective practice**

Self-care and reflective practice were evident in the action learning structures described for nurse and midwife navigators. In the work by Brown et al. [40], navigators participated in repeated action learning groups that encouraged reflection on work experiences, peer support and shared problem-solving. Quantitative measures showed limited change, but qualitative accounts indicated sustained well-being and resilience over time, which the authors linked to the combination of self-care practices and supportive structures for reflection. These findings suggest that, within the JD-R perspective, personal resources were cultivated not only through individual skills training but also through regular, structured opportunities for reflection embedded in team routines.

Only one empirical study in the dataset was conducted in a Gulf region setting. The person-directed programme in Saudi Arabia produced short-term reductions in burnout, with attenuation by six months [33], indicating that personal-resource interventions in this context have been tested only in a limited form and with short follow-up.

**Evidence gaps**

Several gaps in the evidence base were apparent. First, few studies evaluated organisational or team-level strategies that were explicitly designed for nurse case managers; many records focused on adjacent roles such as navigators, care coordinators, and general ward nurses, or described development-stage work without outcome data [34,35,36,37,38,43]. Second, engagement outcomes were measured less frequently than burnout, despite engagement being central to the JD-R motivational process. Third, the durability and scalability of interventions were rarely tested. Where follow-up was available, effects were often short-term or descriptive, as in the partial rebound of burnout after a single two-day programme and the absence of clear quantitative change in longitudinal navigation programmes [33,40]. Fourth, few studies modelled configurations of demands and resources or tested buffering effects, which limits the ability to examine JD-R pathways empirically in real service contexts. Finally, Gulf region evidence was sparse, with only one empirical intervention study from the region, constraining context-specific conclusions for Gulf health systems.

## 4. Discussion

This scoping review aimed to identify organisational and team-level strategies used to enhance work engagement and reduce burnout among nurse case managers and closely aligned roles, and to interpret these strategies using the Job Demands–Resources (JD-R) framework with a view to their applicability in Gulf region health systems. The review adds to the broader nursing literature by focusing on nurse case managers as a distinct group, by systematically mapping strategies to JD-R domains, and by considering how global evidence might inform organisation and team-level interventions in a region where mental health burden and burnout among nurses are well documented.

### 4.1. Interpretation in Relation to the JD-R Framework and Existing Evidence

The JD-R framework proposes that job demands are aspects of work that require sustained effort and incur physiological or psychological costs, whereas job resources are aspects that help staff achieve work goals, reduce demands, or support learning and development; personal resources such as self-efficacy and coping skills are also relevant [8,9,10]. Meta-analytic evidence indicates that job resources are the most proximal drivers of engagement and can buffer the adverse impact of high demands [11], and nursing studies show that job demands are associated with burnout, while job resources are associated with engagement and better work outcomes [12]. A further interpretive point is that the review’s aim centred on work engagement, whereas several included studies assessed burnout or adjacent constructs rather than engagement itself. We therefore treated burnout, well-being, resilience, and job satisfaction as related indicators within a broader JD-R-informed interpretation, while recognising that they do not constitute direct measures of engagement.

Within this framework, the job resources identified in this review are consistent with what is already known about engagement and burnout among nurses, while providing greater specificity for case management roles. Structural enablers and staffing arrangements emerged as foundational resources. Included studies described permanent funding for coordination roles, clear role descriptions and governance structures as prerequisites for sustaining these posts in complex systems. This pattern is consistent with evidence that higher registered nurse staffing is associated with lower mortality and better patient outcomes, while understaffing, high patient-to-nurse ratios, and long or rotating shifts are associated with higher burnout and dissatisfaction [13,14,15]. The finding that churn and vacancy in care manager positions were linked to poorer access outcomes extends this broader literature by highlighting continuity in case management posts as a structural resource for both staff and patients.

Coordination infrastructure and team-based resource building identified in the review overlap closely with organisational and team resources described in previous work. Shared care plans, interoperable information platforms and clear points of contact were described as reducing the friction of cross-setting coordination. This is in line with evidence that effective teamwork and psychological safety support speaking up, learning and coordination, and are associated with improved patient and organisational outcomes and more engaging work environments [18]. Structured team huddles, debriefs, peer consultation rounds, and team charters correspond to features emphasised in Magnet-type work environment programmes, such as participatory decision making, supportive supervision, and professional autonomy, which have been linked to lower burnout, higher satisfaction, and, in several studies, better patient outcomes [19].

The review also highlighted training and supportive supervision, as well as manager–staff development, as central job resources. This accords with evidence that positive leadership styles, including transformational, engaging and caring leadership, are associated with higher nurse engagement and better well-being [16], and that perceived organisational and supervisory support are inversely related to turnover intention and positively related to engagement [17]. Intervention studies showing that job crafting programmes and leadership development can elevate engagement and strengthen unit-level management capabilities further support the view that leadership and supervisory practices are key levers for engagement [21]. Against this background, the emphasis in several included studies on coaching, Appreciative Inquiry cycles and structured supervisor–staff development is coherent with both JD-R and the observational evidence base.

The job demands identified in the review are also consistent with, but more specific than, those described for nurses overall. High caseloads, discharge pressures, cross-sector coordination workload and team churn or vacancy reflect structural demands that are already known to contribute to burnout risks and poorer outcomes in nursing [13,14]. The review adds nuance by emphasising role complexity for nurse case managers, whose responsibilities span assessment, planning, coordination, interprofessional communication, advocacy and documentation across episodes and settings of care [6]. This supports the argument that nurse case managers have a distinct demand profile that is not fully visible when nursing is treated as a unitary occupational category [27]. The accumulation of high coordination workload, accelerated discharges and instability in key posts is consistent with JD- [34] R evidence that multiple demands can create chronic strain when they are not matched by adequate resources [8,11].

The personal-resource strategies in the review were fewer in number and were mainly short stress management and communication skills programmes or reflective practice structures. The quasi-experimental intervention that produced a reduction in burnout at one month, with attenuation by six months, mirrors findings in reviews where mindfulness-based and multimodal programmes yield small to moderate improvements in burnout that are often not sustained in the absence of wider organisational change [22]. Broader healthcare worker reviews report improvements in engagement and resilience following workplace interventions, but highlight methodological limitations and the need for context-specific organisational designs [23]. Taken together, these strands of evidence and the pattern observed in the present review suggest that person-directed strategies can contribute to outcomes, but are unlikely to be sufficient if structural demands remain high and job resources are not strengthened. This is consistent with JD-R formulations that view personal resources as complementing, rather than replacing, job resources in sustaining engagement [9].

Overall, the mapping of resources, demands, and personal strategies for nurse case managers and allied roles accords with broader work, linking higher nurse engagement to stronger patient safety culture, fewer adverse events, and better patient satisfaction, as well as linking burnout to lower quality, reduced safety, and poorer satisfaction [1,3,4,5]. The present synthesis supports the view that engagement among nurse case managers depends on the configuration of structural and team-level resources relative to the specific demands of coordination and navigation work, rather than on individual resilience alone. Across the included studies, the clearest convergence was the recurring importance of structural and relational job resources, particularly staffing stability, coordination infrastructure, supportive supervision, and team-based support. Divergences were most apparent in the measured effects of person-directed interventions and in whether work engagement itself was assessed directly or only through related constructs. Unanswered gaps include the scarcity of case-manager-specific intervention studies, limited follow-up, very sparse Gulf region evidence, and the lack of studies examining how demands and resources operate together over time. From a clinical perspective, these patterns suggest that nurse case managers are unlikely to benefit from resilience-focused programmes alone unless organisations also address workload, coordination processes, and supervisory support. Across the included studies, the clearest pattern was the recurring importance of structural and relational job resources, particularly staffing stability, coordination infrastructure, supportive supervision, and team-based support. Divergence was most evident in the measured effects of person-directed interventions and in the extent to which work engagement was assessed directly rather than through related constructs. These findings suggest that nurse case managers are unlikely to benefit from resilience-focused programmes alone unless organisations also address workload, coordination processes, and supervisory support.

### 4.2. Implications for Practice and Policy, with Attention to Gulf Region Health Systems

The predominance of organisation-directed strategies in the map is consistent with intervention evidence showing that approaches which modify structures, leadership practices, workflows, or staffing can improve workforce outcomes, although effect sizes and durability vary [20]. For nurse case managers, this suggests that efforts to enhance engagement and reduce burnout should prioritise structural enablers, stable and adequate staffing, coordination infrastructure, and leadership and team development, rather than relying mainly on individual-focused programmes. This emphasis aligns with JD-R, which positions job resources as key drivers of engagement and as buffers against the effects of high demands [8,9].

In practical terms, health service leaders can use the JD-R themes identified in this review as a scaffold for designing multi-component bundles. For example, an intervention could combine actions to clarify role scope and secure funding for case manager posts, measures to stabilise staffing and manage caseloads, investments in shared information systems and coordination tools, leadership development to strengthen supportive supervision, and protected time for structured team reflection. Reviews of burnout interventions and organisational strategies in healthcare indicate that such multifaceted, context-sensitive approaches are more likely to be effective than narrow programmes that target only individual coping skills [22,23].

The Gulf region context heightens the importance of these organisational and team-level levers. Syntheses from Gulf Cooperation Council countries report substantial stress, anxiety, depression, and burnout among healthcare workers, including nurses [24]. Multicentre studies from Saudi Arabia and the United Arab Emirates describe notable burnout prevalence among nurses and highlight workload, staffing, and administrative support as salient contributors [25]. Additional work documents high psychological distress, moral distress, and intention to leave among nurses in the region [26]. When these findings are considered alongside the JD-R mapping from this review, it is reasonable to propose that Gulf region health systems may benefit from adapting JD-R-informed bundles of structural, leadership, and team strategies for nurse case managers and similar coordination roles. These proposals are deductions from combined evidence, rather than tested intervention packages, and will require careful co-design, piloting, and evaluation within local regulatory, cultural, and workforce contexts. These implications should therefore be read as hypothesis-generating and practice-informing rather than as definitive recommendations about effectiveness.

### 4.3. Strengths and Limitations of the Evidence Base

This review has several strengths. It focuses on organisational and team-level strategies for nurse case managers and closely aligned roles, and interprets them through a theoretical framework that is well supported in nursing and healthcare [8,11,12]. Mapping interventions explicitly to JD-R domains provided a structured way to organise diverse evidence and to connect the findings to established processes of health impairment and motivation. The review also situates these findings within the specific context of Gulf region health systems, where mental health burden and burnout among nurses are significant concerns [24,25,26].

Important limitations remain. First, few included studies targeted nurse case managers as a clearly defined group; many involved related roles such as navigators, care coordinators, or transitional care nurses, which increases heterogeneity in role definitions and settings [6]. Second, several records were developmental studies, qualitative evaluations, or protocols rather than completed trials, which enhances understanding of mechanisms and implementation challenges but constrains inferences about effect sizes and long-term sustainability [20]. Third, engagement outcomes were seldom measured, despite strong evidence linking engagement to patient safety, service quality and retention [1, 3, 5]. Fourth, only one empirical intervention study originated from a Gulf region health system, which limits context-specific conclusions for that region [33]. This required cautious interpretation of engagement-related conclusions, as some inferences were necessarily based on adjacent rather than direct engagement outcomes. Finally, variation in outcome measures, follow-up periods, and reporting quality hindered cross-study comparison and precluded any formal synthesis of effect sizes. Collectively, these features indicate that the current evidence base is more informative for identifying patterns, mechanisms, and intervention targets than for supporting strong causal or effectiveness claims.

Publication bias and selective reporting are also possible, as studies with null or negative findings may be under-represented. In addition, by restricting inclusion to English language publications and to a defined time period, relevant work in other languages or earlier years may have been missed.

### 4.4. Implications for Future Research

Future research should prioritise evaluations of multi-component JD-R-informed interventions that explicitly target nurse case managers and comparable coordination roles. Designs such as cluster randomised or stepped wedge trials, combined with mixed methods process evaluations, would help clarify how different combinations of structural enablers, staffing arrangements, leadership practices and coordination tools influence job demands, job resources, engagement and burnout over time. To test JD-R mechanisms more rigorously, studies should measure demands and resources alongside engagement and burnout using validated instruments, and should include patient and organisational outcomes such as access, safety events, satisfaction and retention [2,5,13].

In Gulf region health systems, there is a need for co-designed implementation research that adapts and tests JD-R aligned bundles for nurse case managers and related roles, explicitly addressing the high levels of stress, burnout and intention to leave documented among nurses [24,25,26]. Such work should consider local staffing models, regulatory frameworks, cultural expectations regarding leadership and teamwork, and existing digital infrastructure for coordination. Transparent reporting of intervention components, implementation processes, and contextual factors will be essential to build a transferable evidence base.

### 4.5. Summary of Main Findings

In summary, this scoping review shows that organisational and team-level strategies to support nurse case managers and closely aligned roles cluster in predictable ways within the JD-R framework. Job resources in the included studies centred on structural enablers, coordination infrastructure, staffing stability, training and supportive supervision, manager–staff development and team-based resource building. Job demands included role complexity, high caseloads and system tensions, accelerated discharges and disrupted connections, cross-sector coordination workload and team churn or vacancy. Personal resources were targeted mainly through stress management, communication skills and reflective practice. Taken together, the mapped evidence suggests that organisation-directed strategies that strengthen job resources and address chronic demands may offer the most promising route to supporting engagement and limiting burnout among nurse case managers; however, these findings remain exploratory and should be interpreted cautiously in light of the limited and heterogeneous evidence base.

## 5. Conclusions

This scoping review mapped organisational and team-level strategies relevant to work engagement and burnout among nurse case managers and closely aligned roles. Across a limited and heterogeneous evidence base, the most consistent signals related to structural and team-level job resources, particularly staffing stability, coordination infrastructure, supportive supervision, and team-based support, while person-directed interventions appeared narrower in scope and less durable in effect. Direct evidence from Gulf region health systems was scarce.

Overall, the review suggests that strengthening organisational resources and addressing chronic job demands may offer the most promising route to supporting engagement and mitigating burnout in nurse case management roles. These findings are exploratory and should guide context-sensitive intervention design, co-design, and evaluation rather than be interpreted as definitive evidence of effectiveness.

## Figures and Tables

**Table 1:** PCC eligibility matrix with inclusion and exclusion criteria.

Domain	Inclusion Criteria	Exclusion Criteria
Population	Registered nurse case managers and aligned roles: nurse navigators, care coordinators, discharge planners, utilisation review nurses.	Studies not involving nursing or healthcare; roles without case management or coordination functions.
Concept	Organisational or team-level strategies, policies, programmes, or practice changes to enhance work engagement or reduce burnout; studies that report job demands/resources linked to engagement or burnout to enable JD-R mapping.	Items that do not describe or recommend any strategy or intervention.
Context	Any healthcare setting worldwide; subgroup mapping for Gulf region health systems.	Non-healthcare contexts.
Study designs	Qualitative, quantitative, mixed-methods empirical studies; evidence syntheses; conceptual papers that specify actionable strategies.	Editorials, opinion pieces, and commentaries without analysable data.
Timeframe	Publications dated 1 January 2015 to 31 December 2025.	Items published before 2015.
Language	English.	Non-English.
Outcomes/Focus	Engagement, burnout, job demands, job resources, or strategic interventions related to these constructs.	No engagement/burnout focus and no JD-R linkage.
Availability	Full text available.	Abstract only; inaccessible full text.

**Table 2:** Characteristics of included studies, charted.

Study (Year) & Region	Population and Setting	Design and Instruments	JD-R Mapped Exposures/Interventions	Primary Outcomes	Key Findings (Direction and Effect)	Role Relevance
“Burning out physical and emotional fatigue: Evaluating the effects of a programme aimed at reducing burnout among mental health nurses”. Alenezi et al. [33] (Saudi Arabia)	Mental health nurses in hospital units	Quasi-experimental (non-equivalent groups); 2-day workshop; Maslach Burnout Inventory at baseline, 1, 3 and 6 months	Person-directed programme focusing on stress management and communication or social skills (personal resources)	Burnout (MBI emotional exhaustion, depersonalisation, and personal accomplishment)	Burnout scores decreased at 1 month and partially returned toward baseline by 6 months	General hospital nurses: role adjacent to nurse case managers
“Building and sustaining work engagement—a participatory action intervention to increase work engagement in nursing staff”. Knight et al. [34] (UK)	Nursing staff on older people’s acute wards	Non-randomised matched pre–post design with controls; participatory-action intervention; Utrecht Work Engagement Scale; and basic psychological needs, job demands and resources measures	Team-based resource building to enhance social support and decision influence (job resources)	Work engagement; autonomy, competence, and relatedness; job demands and job resources	No significant improvement in work engagement; relatedness decreased, and competence showed borderline decrease; mediation analysis supported JD-R and self-determination theory pathways; implementation readiness was reported as important	General ward nurses: role adjacent to nurse case managers
“Development of a nurse–manager dualistic intervention programme to alleviate burnout among nurses based on Appreciative Inquiry”. Guo et al. [35] (China)	Registered nurses in a hospital context	Programme development study; JD-R guided nurse manager dualistic intervention; six sessions across nine weeks using Appreciative Inquiry	Combined person and organisation intervention focusing on manager staff development (job resources and personal resources)	Intended: burnout, job resources, work engagement (not yet measured)	Development phase only; content and structure of the programme were specified; no outcome data available at the time of reporting	General nurses: role adjacent to nurse case managers
“The Value of the Nurse Navigator in Complex Cancer Care: A Scoping Review”. Muzio et al. [36] (International/Canada)	Nurse navigators in public cancer programmes	Scoping review (44 studies)	Role and organisational components related to coordination, advocacy, education, psychosocial support, assessment and workflow (job resources and job demands)	Patient, provider, and system-level outcomes	Reported benefits included more patient-centered care, improved support for providers and better coordination; reported barriers included work overload and inefficient workflows; recommendations included integrated teams and permanent funding for navigator roles	Navigator role: analogous to nurse case managers
“Effects of interventions aimed at improving nurses’ work engagement in the workplace: a systematic review and meta-analysis protocol”. Kuribayashi et al. [37] (Japan)	Nurses across healthcare settings	Systematic review and meta-analysis protocol for randomised controlled trials of psychosocial interventions; Utrecht Work Engagement Scale planned	Planned resource building, leadership and health promotion interventions (job resources and personal resources)	Work engagement (planned)	Protocol only; no intervention effects reported; identifies the evidence gap on nurse-specific work engagement interventions	General nurses: role adjacent to nurse case managers
“Challenges and Adaptive Strategies in Transitional Care During COVID-19: A Qualitative Study of Nurses’ Experiences in Japan”. Sumikawa et al. [38] (Japan)	Transitional care nurses in acute hospitals	Qualitative descriptive study; semi-structured interviews with 15 nurses	Observed hybrid workflows and community partnerships; demands included disrupted connections and accelerated discharges (job demands and job resources)	Experience themes	Described disrupted connections between hospital and community, professional pressures and adaptive strategies; hybrid models were perceived as supporting continuity of care under pandemic conditions	Transitional care coordinators with roles similar to nurse case managers
“Care Coordinators in Integrated Care: Burnout Risk, Perceived Supports, and Job Satisfaction”. Au et al. [39] (USA)	Care coordinators in integrated physical and behavioural healthcare	Mixed methods study with surveys, interviews and focus groups (n = 231); Maslach Burnout Inventory	Demands related to complex cross-sector coordination; resources included training, supportive supervisors and team support (job demands and job resources)	Burnout risk; job satisfaction; perceived supports	Overall burnout risk was low; participants reported high levels of organisational, supervisory and team support; supports were described as protective in relation to role demands	Care coordinators with roles similar to nurse case managers
“Nurse and midwife navigator resilience, well-being, burnout, and turnover intent: A multi-methods study”. Brown et al. [40] (Australia and New Zealand)	Nurse and midwife navigators in public hospitals	Longitudinal multi-method study (five time points) with action learning groups	Work-based supports, leadership and governance and self-care practices; demands included high role complexity and cross-sector coordination (job resources, personal resources and job demands)	Resilience, well-being, burnout, turnover intention	Quantitative indicators showed no significant change over time; qualitative data indicated sustained well-being and resilience, which participants linked to leadership support, governance structures and action learning groups	Navigator role: analogous to nurse case managers
“Churning the tides of care: when nurse turnover makes waves in patient access to primary care”. Arredondo et al. [41] (United States Veterans Health Administration)	Registered nurse care managers on patient-aligned care teams across 152 facilities	Retrospective database study over 24 months; 5897 teams	Exposure was RN care manager role stability versus churn or vacancy; resources included adequate full-time equivalent and team size (job resources and job demands)	Access metrics (time to next available appointment, wait times, urgent care or emergency use, patient messaging)	Teams with continuous churn or vacancy in RN care manager positions had longer waits and more urgent care or emergency use; teams with stable RN care manager full-time equivalent were associated with better access metrics	RN care managers with roles similar to nurse case managers
“Facilitating and barrier factors to the implementation of a transitional care programme: a qualitative study of hospital coordinators in South Korea”. Park et al. [42] (South Korea)	Hospital coordinators, nurses and social workers in a congenital long-term disease programme	Qualitative interview study with 41 participants	Facilitators included cooperative relationships, shared information technology platform and institutional support; demands included cross-institution coordination workload (job resources and job demands)	Implementation facilitators and barriers	Identified the need for a shared information platform and institutional assistance to support coordination; heavy cross-institutional coordination workload and limited capability were reported as barriers	Hospital-based transitional care roles similar to nurse case managers
“Implementing and sustaining dementia care coordinators across integrated care systems: a realist evaluation”. Abrams et al. [43] (UK)	Dementia care coordinators across 42 primary care networks	Realist evaluation with repeat survey and 57 interviews; included Maslach Burnout Inventory	Demands included increasing caseloads and system tensions; resources included workforce design, connections, training, funding and a single point of contact (job demands and job resources)	Implementation outcomes and burnout risk	Coordinators were described as bridge builders between services; growth in caseloads and system tensions raised concerns about sustainability and potential burnout; authors recommended attention to funding, workload and ongoing monitoring	Dementia care coordinator role: similar to nurse case managers

Note: Included records comprised both empirical and non-empirical/developmental contributions; outcome-related inferences were drawn primarily from empirical studies.

**Table 3:** JD-R mapping of demands, resources, and sub-themes.

JD-R Domain (Theme)	Sub-Theme	Operational Examples	Studies	Count (Studies)
Job resources	Structural enablers	Secure multi-year funding lines for navigator and case-manager posts; formalise role descriptions and scope; establish governance committees for coordination services; create job ladders with progression criteria; and introduce workload weighting in schedules to reflect case complexity.	[36,42,43]	3
Job resources	Coordination infrastructure	Deploy a single shared care plan within the electronic health record; implement an interoperable referral and appointment system; standardise handover templates for transfers between facilities; schedule inter-agency case conferences; and designate a single point of contact for patients and families.	[42,43]	2
Job resources	Staffing stability and adequate full-time equivalent staffing	Set minimum nurse case manager to patient ratios; create a backfill pool to cover vacancies and leave; cap caseloads using complexity weighting; offer retention and re-entry programmes; and shorten vacancy periods through fast-track recruitment.	[41]	1
Job resources	Training and supportive supervision	Provide communication and de-escalation skills workshops; deliver structured stress management sessions with planned booster sessions; schedule supervisor-led reflective practice groups; offer orientation programmes for new case managers; and train supervisors in supportive behaviours and workload triage.	[37,39]	2
Job resources	Manager-staff development	Implement coaching for frontline supervisors; schedule monthly one-to-one meetings focused on support and workload; deliver Appreciative Inquiry cycles for manager–staff problem-solving; pair new case managers with senior mentors; provide short learning modules on giving feedback and recognising contributions.	[35,40]	2
Job resources	Team-based resource building	Establish regular multidisciplinary huddles for complex cases; protect time for team debriefs after high-pressure days; adopt shared decision-making protocols at the unit level; run peer consultation rounds for difficult discharges; and create a team charter that clarifies roles and escalation paths.	[34,39,40,43]	4
Job demands	Role complexity	Cover broad and shifting responsibilities across specialties; switch frequently between coordination, education, and administrative tasks; and work within unclear scope boundaries that require constant negotiation.	[40]	1
Job demands	High caseloads and system tensions	Maintain caseloads above agreed thresholds without relief; set conflicting targets that prioritise throughput over coordination quality; and manage bed shortages that force rapid turnover and compress coordination time.	[43]	1
Job demands	Accelerated discharges and disrupted connections	Meet fixed discharge deadlines that outpace home-care readiness; discharge patients before care plans are complete; and encounter gaps in post-discharge follow-up due to limited capacity.	[38]	1
Job demands	Cross-sector coordination workload	Re-enter data across multiple systems; obtain external approvals without standard memoranda of understanding; arrange community services when contact points are unclear; and repeatedly chase information from providers outside the network.	[38,39,40]	3
Job demands	Team churn or vacancy	Leave nurse case-manager positions unfilled for extended periods; rely on rotating temporary staff; and reassign staff frequently, disrupting continuity with patients and teams.	[41]	1
Personal resources	Stress management and communication skills	Deliver brief courses on stress coping and cognitive reframing; provide training in structured communication for difficult conversations; integrate short mindfulness practices at the start of shifts; and run workshops on resolving conflict.	[33]	1
Personal resources	Self-care and reflective practice	Schedule regular reflective huddles; establish peer-support circles or action-learning sets; debrief after critical incidents using a standard format; and use personal development plans linked to supervisory meetings.	[40]	1

## Data Availability

Data sharing is not applicable to this article as no new data were created or analysed in this study. All data included in this review are derived from previously published studies, which are cited in the reference list.

## Supplementary Materials

The following supporting information can be downloaded at: https://www.mdpi.com/article/10.3390/nursrep16040145/s1, Table S1. Full search strategies for all databases.

## Public Involvement Statement

No public involvement in any aspect of this research.

## Guidelines and Standards Statement

This manuscript was drafted in accordance with the PRISMA-ScR (Preferred Reporting Items for Systematic Reviews and Meta-Analyses Extension for Scoping Reviews) guidelines for scoping review research, and the search strategy was reported in line with PRISMA-S.
