# Supplementary material for: Organisational and Team-Level Strategies to Enhance Work Engagement and Mitigate Burnout Among Nurse Case Managers: A Global Scoping Review with Implications for the Gulf Region
Source: Nurs Rep. 2026 Apr 17;16(4):145. doi: 10.3390/nursrep16040145 (PMC13118688; doi:10.3390/nursrep16040145)
Supplement: Supplementary file 1 [file nursrep-16-00145-s001.zip › nursrep-4243926-supplementary.pdf]

Supplementary Table S1. Full search strategies for all databases.

Database	Search String/Syntax
PubMed	(“nurse case managers”[Title/Abstract] OR “nursing case managers”[Title/Abstract]) AND (“job demands”[Title/Abstract] OR “workload”[Title/Abstract] OR “emotional labor”[Title/Abstract]) AND (“job resources”[Title/Abstract] OR “organizational support”[Title/Abstract] OR “resilience”[Title/Abstract]) AND (“burnout, professional”[MeSH] OR “burnout”[Title/Abstract] OR “emotional exhaustion”[Title/Abstract]) AND (“work engagement”[Title/Abstract] OR “occupational engagement”[Title/Abstract]) AND (“healthcare setting”[Title/Abstract] OR “hospital”[Title/Abstract])
CINAHL	(“nurse case manager*” OR “case management nurse*”) AND (“job demands” OR workload OR “emotional labor”) AND (“job resources” OR “organizational support” OR “autonomy”) AND (“burnout” OR “emotional exhaustion”) AND (“work engagement” OR “employee engagement”) AND (“healthcare setting*” OR hospital*)
PsycINFO	(“nurse case manager*” OR “case management nurse*”) AND (“job demands” OR “role conflict” OR workload) AND (“job resources” OR “autonomy” OR “organizational support”) AND (“work engagement” OR “employee engagement”) AND (“burnout” OR “occupational stress”) AND (“healthcare setting*” OR “hospital*” OR “nursing environment”)
Scopus	TITLE-ABS-KEY(“nurse case manager*” OR “nursing case manager*”) AND TITLE-ABS-KEY(“job demands” OR “workload” OR “emotional labor”) AND TITLE-ABS-KEY(“job resources” OR “organizational support” OR “resilience”) AND TITLE-ABS-KEY(“work engagement” OR “employee engagement”) AND TITLE-ABS-KEY(“burnout” OR “emotional exhaustion”) AND TITLE-ABS-KEY(“healthcare setting*” OR “hospital*” OR “clinical environment”)
Web of Science	TS = (“nurse case manager*” OR “case management nurse*”) AND TS = (“job demands” OR workload OR “emotional labor”) AND TS = (“job resources” OR “organizational support” OR autonomy) AND TS = (“burnout” OR “emotional exhaustion”) AND TS = (“work engagement” OR “occupational engagement”) AND TS = (“healthcare setting*” OR hospital*)
Google Scholar	“nurse case manager” AND (“job demands” OR “workload” OR “emotional labor”) AND (“job resources” OR “organizational support” OR “resilience”) AND (“work engagement” OR “employee engagement”) AND (“burnout” OR “emotional exhaustion”) AND (“healthcare setting” OR hospital OR “nursing environment”)
